# Works Following

**Published:** 1870-08

**Authors:** 


### WORKS FOLLOWING.

THE memory of the good is blessed: and this is written as an encouragement to the good, and as a reminder to those benefited, that long after kindness has been done there ought to be acknowledgments of it; for thereby happy feelings are excited in the minds of those who give, and those who receive; and who shall say that such things do not add to the value and the length of human life? and what a different world this would be, if every one would consider it a day lost in which some kind thought has not been had of others, or some kind word said, or some kind deed done! And how much worse than a loss is it to the unfortunate of our kind, who can scarcely ever retire to their couch at night without having occasion for remorse at the remembrance of spiteful words said, of vindictive feelings experienced, and hard, harsh acts done towards others. A handsome remembrance of an eminent name, thought of with affectionate respect by many a New Yorker, is thus recorded: —

The Rev. W. A. Scott, D. D., of San Francisco, received recently a very handsome tribute of affection from " the young men of the Sabbath School of the Forty-second Street Presbyterian Church," New York, of which he was formerly pastor. This token of kind remembrance, is a copy of the complete works of St. Augustine, the great Latin Father of the Church. It is in sixteen royal octavo volumes, double columns, and handsomely bound. It is from the Paris edition of the *Patrologiæ Latinea of Migne*, Paris, 1865. It was imported by order from Europe expressly for Dr. Scott.

On Dr. Scott's arrival at San Francisco, a handsome church edifice was purchased and beautified for him at an expense of a hundred thousand dollars, and we trust that there, with his children and grandchildren gathered around him, he may be permitted after many more years of health and usefulness, to close a life of remarkable industry, efficiency, and success in the great vineyard of the Master.

